# Construction and Verification of a Predictive Model for Risk Factors in Children With Severe Adenoviral Pneumonia

**DOI:** 10.3389/fped.2022.874822

**Published:** 2022-06-27

**Authors:** Yaqiong He, Peng Liu, Leyun Xie, Saizhen Zeng, Huashan Lin, Bing Zhang, Jianbin Liu

**Affiliations:** ^1^Department of Radiology, Hunan Provincial People’s Hospital (The First Affiliated Hospital of Hunan Normal University), Changsha, China; ^2^Department of Pediatrics, Hunan Provincial People’s Hospital (The First Affiliated Hospital of Hunan Normal University), Changsha, China; ^3^GE Healthcare, Changsha, China

**Keywords:** children, adenovirus pneumonia, low-dose CT, nomogram, prediction model

## Abstract

**Objective:**

To construct and validate a predictive model for risk factors in children with severe adenoviral pneumonia based on chest low-dose CT imaging and clinical features.

**Methods:**

A total of 177 patients with adenoviral pneumonia who underwent low-dose CT examination were collected between January 2019 and August 2019. The assessment criteria for severe pneumonia were divided into mild group (*N* = 125) and severe group (*N* = 52). All cases divided into training cohort (*N* = 125) and validation cohort (*N* = 52). We constructed a prediction model by drawing a nomogram and verified the predictive efficacy of the model through the ROC curve, calibration curve and decision curve analysis.

**Results:**

The difference was statistically significant (*P* < 0.05) between the mild adenovirus pneumonia group and the severe adenovirus pneumonia group in gender, age, weight, body temperature, L/N ratio, LDH, ALT, AST, CK-MB, ADV DNA, bronchial inflation sign, emphysema, ground glass sign, bronchial wall thickening, bronchiectasis, pleural effusion, consolidation score, and lobular inflammation score. Multivariate logistic regression analysis showed that gender, LDH value, emphysema, consolidation score, and lobular inflammation score were severe independent risk factors for adenovirus pneumonia in children. Logistic regression was employed to construct clinical model, imaging semantic feature model, and combined model. The AUC values of the training sets of the three models were 0.85 (0.77–0.94), 0.83 (0.75–0.91), and 0.91 (0.85–0.97). The AUC of the validation set was 0.77 (0.64–0.91), 0.83 (0.71–0.94), and 0.85 (0.73–0.96), respectively. The calibration curve fit good of the three models. The clinical decision curve analysis demonstrates the clinical application value of the nomogram prediction model.

**Conclusion:**

The prediction model based on chest low-dose CT image characteristics and clinical characteristics has relatively clear predictive value in distinguishing mild adenovirus pneumonia from severe adenovirus pneumonia in children and might provide a new method for early clinical prediction of the outcome of adenovirus pneumonia in children.

## Introduction

Adenovirus (ADV) pneumonia is one of the more community-acquired severe pneumonia in children. Adenovirus infections are more common in young children, specifically in 6-month to 2-year-old infants owing to a lack of humoral immunity (1). Severe adenoviral pneumonia has a rapid onset, severe disease, rapid progress, and a poor prognosis (2). Severe adenoviral pneumonia has a high child mortality rate and a long recovery period, easily linked to multiple organ damage. The mortality rate of untreated severe adenovirus pneumonia or other communicable diseases may exceed 50%. Severe lung lesions, acute respiratory distress syndrome (ARDS), and severe coagulation abnormalities are the direct causes of death in children. 14–16% of recovered patients may have different degrees of sequelae (3). Occlusion Bronchitis and bronchiectasis are the most common complications in children with severe adenoviral pneumonia. We have encountered some patients with adenoviral pneumonia with mild clinical symptoms who rapidly develop respiratory failure in our clinical practice. Consequently, identifying risk factors early and reminding clinicians to implement intervention strategies as soon as possible is critical. The imaging changes of children with adenovirus pneumonia appear early. The imaging examinations are crucial for diagnosing and condition judgment of adenovirus pneumonia. At present, clinical radiology diagnosis reports primarily rely on assessing the severity of lung infiltration, which is highly subjective and lacks quantitative parameter indicators and related standards. This study aimed to use the lung consolidation scores, lobular inflammation scores, and assessment of small airway lesions, combined with clinical data and laboratory examinations, to establish a predictive model for risk factors with severe adenovirus pneumonia in children.

## Materials and Methods

### Patient Information

**FIGURE 1:**
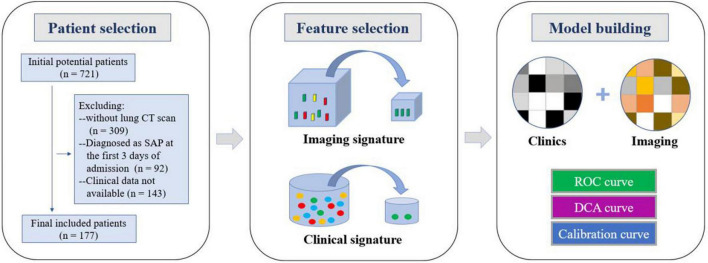
Workflow of the study. Workflow can be divided into three parts:patient selection, feature extraction, and model construction.

The study protocol was approved by the Ethical Review Committee of Hunan People’s Hospital. The parents or guardians of all of the participants in this cooperative study provided written informed consent. This study retrospectively analyzed 721 patients with adenovirus pneumonia hospitalized in Hunan Provincial People’s Hospital from January 2019 to December 2019. They were excluded if they had congenital lung dysplasia, airway malformations, congenital immunodeficiency, congenital heart disease, malnutrition, or congenital metabolic syndrome disease and 177 children were enrolled after screening (Figure 1). Inclusion criteria were: (1) patients who met the diagnostic criteria for adenovirus pneumonia and the clinical diagnosis of admission had mild pneumonia and did not develop severe pneumonia within 3 days of admission; (2) a low-dose chest CT examination was performed within 3 days of admission, and the image met the diagnostic criteria; and (3) complete clinical data were collected. Exclusion criteria were: (1) patients who did not have a low-dose chest CT examination (*n* = 309); (2) patients who were diagnosed with severe childhood adenovirus pneumonia within 3 days of admission (*n* = 92); and (3) patients whose clinical data are not available (*n* = 143). Patients were enrolled after diagnosed with adenovirus. Nasopharyngeal swab sampling results and clinical signs were collected, including gender, age, weight, body temperature at admission, respiratory rate, heart rate, family history, vaccination status data, and experiments on admission. Laboratory examination data and detailed records, including blood routine, immune factors, sputum culture, adenovirus typing, and virus copy number were collected by the same time.

The patients were divided into mild adenovirus pneumonia and severe adenovirus pneumonia. The criteria were based on the Chinese 2019 version of diagnosing and treating children’s community-acquired pneumonia (4). Severe pneumonia was diagnosed when any of the following criteria were met. (1) Overall poor health condition of a patient. (2) A conscious disturbance. (3) Cyanosis or tachypnea [age < 2 months: respiratory rate (RR) 60 breaths/minute; age 2 months to 1 year: RR 50 breaths/minute; age 1–5 years: RR 40 breaths/minute; and age > 5 years: RR 30 breaths/minute)], intermittent apnea, or oxygen saturation of < 92%. (4) Persistent hyperpyrexia or ultrahyperpyrexia for more than 5 days. (5) Dehydration or food refusal. (6) A chest X-ray or CT reveals pulmonary infiltration of at least two-thirds of the lung, pneumothorax, lung necrosis, or lung abscess on one side. (7) Extrapulmonary complications.

The diagnostic criteria for adenoviral pneumonia were based on the “Diagnosis and Treatment Criteria for Adenoviral Pneumonia in Children (2019 Edition)” promulgated by the National Health Commission of China and the State Administration of Traditional Chinese Medicine in this study (5). The diagnostic criteria for ADV were based on ADV antigen positivity as determined by a nasopharyngeal swab or alveolar lavage fluid test, followed by an immunofluorescence test or a nucleic acid test.

### Lung CT Examination

A total of 177 children underwent low-dose chest CT scans using NeuViz 64 En CT (Shenyang Neusoft Medical System Co., Ltd.) or Philips 256 Brilliance iCT (Philips, Netherlands). The voltage was 100 KV, the tube current was 30–50 mA, the reconstruction layer thickness was 1.25 mm, and the layer spacing was 1.25 mm.

### CT Image Analysis

Two senior diagnosticians (with 21 and 11 years of experience in chest imaging diagnosis) evaluated all imaging data. The standard of chest CT examination results was based on the priority definition of the “Fleischner Society Glossary” (6), including assessment of lung consolidation and extent of involvement, lobular inflammation and extent of involvement, assessment of small airway disease, including emphysema, mosaic sign, bronchial wall thickening, and bronchiectasis, and whether there is pleural effusion, pneumothorax, emphysema mediastinum, and other extrapulmonary complications. On the CT image, the lungs were divided into six regions: upper (above the carina), middle (below the carina to above the inferior pulmonary vein), and inferior (below the inferior pulmonary vein) (7, 8). Each area was classified as lung consolidation or lobule Inflammation involvement range assignment (9). Each area was scored from 0 to 4 to evaluate the degree of pneumonia involvement in each lung area; 0 was normal, four was the area where all the lung parenchyma was involved. The values of the six areas were accumulated, and the total CT score was between 0–24.

### Statistical Methods

This study’s measurement data have been expressed as mean ± standard deviation or median (upper quartile, lower quartile). A chi-square test or Fisher’s exact test has been used for categorical variables and continuous variables that conform to a normal distribution and have been used in *t*-test, Mann–Whitney test for abnormally distributed continuous variables, univariate analysis of the influencing factors of imaging parameters, multivariate logistic analysis of clinical signs and laboratory test indicators, and multivariate analysis of independent influencing factors, according to the stepwise selection method. The selected method has been incorporated into multivariate analysis. The receiver operating characteristic (ROC) curve has been used to determine the performance of the machine learning model, and the accuracy, sensitivity, specificity, and area under the curve (AUC) are calculated. All statistical analyses were performed using IPMs (GE Healthcare, United States) R (version 3.5.1). A two-tailed *p*-value < 0.05 indicated statistical significance.

The imaging and clinical data of the mild and severe groups were analyzed and compared, using univariate analysis to extract the selected imaging and clinical features. Multivariate logistic analysis was used to conduct multivariate analysis of independent influencing factors. The selected method was incorporated into a multi-factor analysis to screen out the image characteristics and clinical characteristics. Finally, these characteristics were used to establish a prediction model for severe childhood adenovirus pneumonia patients using logistic regression. The risk factors were combined to construct a predictive model, and a nomogram was developed using R language rms package. The enrolled patients were divided into a prediction group and a verification group, and the receiver operating characteristic (ROC) curve was used to determine the performance of the machine learning model, calculate the accuracy, sensitivity, specificity, and area under the curve (AUC), and test the stability of the prediction model for sex and reliability. The calibration curve was used to detect the fit of the model, and DCA evaluated the clinical benefit of patients (Figure 1).

## Results

### General Situation

Table 1 shows the detailed information of patients. Among the 177 patients enrolled in the group, 125 (70.6%) were in the mild group and 52 (29.4%) were in the severe group; 149 were males, including 117 (78.5%) in the mild group and 32 (21.5%) in the severe group; 28 were females, including 8 (28.6%) in the mild group and 20 (71.4%) in the severe group. The number of male cases was significantly higher than that of females, but most of them were in the mild group, and fewer progressed to the severe group; there were fewer female cases in the mild group, but more progressed to the severe group. The average age of onset in the mild group was 36 months (18.1, 55.3), 12 months longer than the average age of onset in the severe group (12, 36). The average weight of the mild group was 15 kg (11.0, 18.1), which was higher than the average weight of the severe group by 12 kg (9.2, 14.4).

**TABLE 1:** Demographic, clinical and chest CT findings of patients with adenovirus pneumonia included in the study.

Characteristics	Total (*n* = 177)	MAP group (*n* = 125)	SAP group (*n* = 52)	*P*-value^a^	Training Cohort (*n* = 125)	Validation Cohort (*n* = 52)	*P*-value^b^
**Demographics**							
Gender				<0.001			0.919
Male	149	117	32		105	44	
Female	28	8	20		20	8	
Age (month)	177	36 (18.1, 55.3)	12 (12, 36)	<0.001	36 (12, 48)	24 (12, 48)	0.908
Weight	177	15 (11.0, 18.1)	12 (9.2, 14.4)	<0.001	14 (10.5, 16.7)	13 (10.7, 17.9)	0.914
**Clinical features**							
Fever (°C)^c^	177	37.1 (36.6, 38.3)	37.9 (37.0, 38.7)	<0.001	37.4 (36.8, 38.4)	37.2 (36.6, 38.5)	0.715
Fever course (days)^d^	177	5 (4, 7)	5 (4, 7)	0.988	5 (4, 7)	5 (4, 6)	0.895
**Laboratory detection**							
WBC (×10 ^9^/L)	177	7.58 (5.17, 10.31)	6.32 (4.8, 9.6)	0.169	6.67 (4.94, 9.99)	7.54 (5.38, 10.06)	0.629
NEU	177	3.34 (2.33, 5.38)	4.57 (3.17, 8.01)	0.005	3.55 (2.63, 6.57)	3.62 (2.37, 5.91)	0.842
Lymphocyte	177	2.73 (1.83, 3.84)	1.98 (1.33, 4.54)	0.168	2.68 (1.69, 4.14)	2.34 (1.66, 3.53)	0.66
L/N ratio	177	0.66 (0.48, 1.26)	0.48 (0.35, 0.84)	0.002	0.65 (0.36, 1.05)	0.66 (0.47, 1.17)	0.591
L/W ratio	177	0.36 (0.29, 0.50)	0.32 (0.25, 0.51)	0.36	0.36 (0.27, 0.51)	0.36 (0.29, 0.49)	0.929
CRP	177	12.8 (4.3, 28.3)	12.5 (4.1, 18.9)	0.3	12.60 (4.33, 24.14)	15.70 (4.06, 33.81)	0.213
LDH	177	343 (285, 425)	629 (423, 989)	<0.001	393 (306, 605)	34 (298, 509)	0.187
ALT	177	15.7 (11.8, 21.3)	22.1 (16.5, 32.0)	<0.001	16.9 (12.8, 25.2)	18.1 (11.7, 24.0)	0.766
AST	177	40.9 (31.1, 58.1)	66.2 (47.3, 102.6)	<0.001	46.9 (34.9, 69.8)	43.6 (32.5, 63.9)	0.386
CK-MB	177	24 (18, 32)	38 (23, 53)	<0.001	28 (20, 41)	23.5 (17.5, 34.7)	0.100
ADV DNA level^e^	177	5.76 (3.96, 7.26)	6.62 (5.66, 7.58)	0.017	6.31 (4.65, 7.40)	5.58 (3.92, 7.07)	0.07
**Imaging characteristics**							
Air bronchogram				0.003			0.193
Positive	78	46	32		59	19	
Negative	99	79	20		66	33	
Pulmonary emphysema				<0.001*			0.190
Positive	85	48	37		64	21	
Negative	92	77	15		61	31	
Mosaic sign				0.462			0.978
Positive	44	33	11		31	13	
Negative	133	92	41		94	39	
Bronchial wall thicken				0.024			0.005
Positive	150	101	49		112	38	
Negative	27	24	3		13	14	
Bronchiectasis				0.608			0.905
Positive	8	5	3		5	3	
Negative	169	120	49		120	49	
Pleural effusion				0.009*			0.987
Positive	12	4	8		9	3	
Negative	165	121	44		116	49	
Consolidation score	177	0 (0, 1)	3 (1, 4)	<0.001*	1.00 (0.00, 2.00)	0.00 (0.00, 2.00)	0.13
Lobular inflammation score	177	3 (1, 5)	4 (2, 6)	0.014*	3.00 (1.00, 5.00)	2.00 (1.00, 4.00)	0.051

*Categorical variables are presented as number of patients and data in parentheses are percentages; continuous variables are showed as median (lower quartile, upper quartile).*

*Statistical analysis was performed with Wilcoxon Test or Fisher exact test.*

The symbol * for P < 0.05 suggests a significant difference.

*^a^Comparing the group with ARDS and the group without ARDS.*

*^b^Comparing between the training cohort and the validation cohort.*

*^c^The temperature of fever was obtained at admission.*

*^d^Fever course was defined as the duration from onset to the day of admission.*

*^e^The data of ADV DNA level underwent a transition of log function.*

*MAP, mild adenovirus pneumonia; SAP, severe adenovirus pneumonia; WBC, white blood cell; NEU, neutrophile; CRP, C-reactive protein; LDH, lactate dehydrogenase; ALT, alanine transaminase; AST, aspartate transaminase; CK-MB, creatine kinase-MB.*

### Clinical Signs and Laboratory Examinations

All the patients in the group suffered from fever. The average body temperature of the mild group was 37.1°C (36.6, 38.3), which was lower than the severe group’s 37.9°C (37.0, 38.7); the fever duration between the mild and severe groups was 5 days, and there was no significant difference (Table 1).

In the mild disease group, the lymphocyte/neutrophil ratio (L/N ratio), lactate dehydrogenase (LDH), alanine transaminase (ALT), asparagus aspartate aminotransferase (AST), creatine kinase-MB form (CK-MB), and ADV DNA levels, as well as White Blood Cell Count (WBC) and neutral hemoglobin, were significantly lower than those in the severe group. There was no significant difference in the levels of neutrophil (NEU), lymphocyte, lymphocyte/white Blood Cell ratio (L/W ratio) and C-reactive protein (CRP) (Table 1).

### Imaging Performance

**FIGURE 2:**
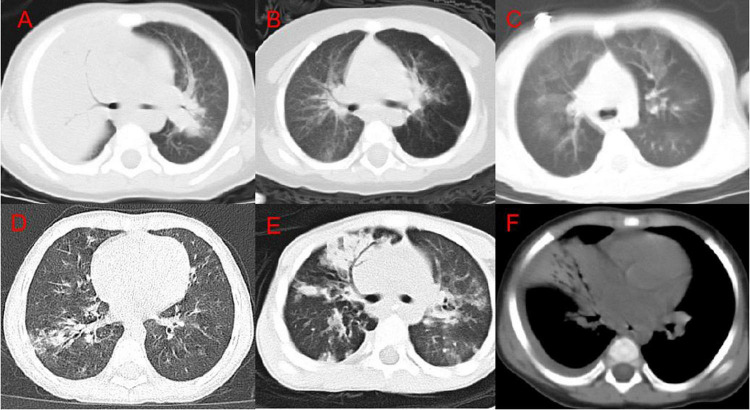
Chest CT imaging findings of adenovirus pneumonia. **(A)** Right upper lung consolidation with bronchial inflation sign; **(B)** left emphysema; **(C)** double lung mosaic sign; **(D)** right lower lung bronchial wall thickening and surrounding lobule inflammation; **(E)** right Middle lung bronchiectasis; **(F)** a small amount of pleural effusion on the right.

Through the analysis of differences, meaningful features have been identified. The symptoms of bronchial inflation, emphysema, ground glass sign, bronchial wall thickening, bronchiectasis, and pleural effusion in the mild group differed from those in the severe group (Table 1). The consolidation score of 0 (0, 1) in the mild group was significantly lower than the consolidation score of 3 (1, 4) in the severe group (*P* < 0.001). The lobular inflammation score of the mild group was significantly lower than the severe group (*P* = 0.014). A total of 78 patients in the enrolled group had bronchial inflation signs (Figure 2A); among them, 46 patients (36.8%) were in the mild group and 32 patients (61.5%) were in the severe group. 85 patients had emphysema (Figure 2B). Among them, 48 cases were in the mild group (38.4%) and 37 cases were in the severe group (71.2%). The incidence of emphysema in the mild group was significantly lower than the severe group (*P* < 0.001). 44 patients had a mosaic sign (Figure 2C). Among them, 33 patients (26.4%) were in the mild group and 11 patients (21.2%) were in the severe group. 150 patients had bronchial wall thickening (Figure 2D); among them, 101 patients (80.8%) were in the mild group and 49 patients (94.2%) were in the severe group. 8 patients had bronchiectasis (Figure 2E), of which five patients (4.0%) were in the mild group and 3 patients (5.8%) were in the severe group. 12 patients had pleural effusion (Figure 2F); among them, the mild group had 4 cases (3.2%) and 8 cases (15.4%) were in the severe group. The incidence of pleural effusion in the mild group was significantly lower than that in the severe group (*P* = 0.009).

### Multivariate Logistic Regression Analysis

A multivariate logistic analysis analyzed clinical signs, laboratory test indicators, and independent factors. The multivariate analysis was incorporated according to a stepwise selection method. The results showed that gender, LDH value, emphysema, consolidation score, and lobular inflammation score were independent risk factors for predicting adenovirus pneumonia in children with a severe health condition (all *P* < 0.05) (Table 2).

**TABLE 2:** Multivariable logistic regression for nomogram construction.

Characteristics	Odd ratio	95% CI	*P*-value
Gender^a^	0.21	0.05–0.83	0.026*
LDH	1.00	1.00–1.00	0.013*
Pulmonary emphysema^b^	8.28	2.23–30.75	0.001*
Consolidation scores	1.53	1.08–2.17	0.017*
Lobular inflammation scores	1.14	0.97–1.34	0.001*

*^a^Male was denoted as 1, and female as 0.*

*The odd ratio was 0.21 means that male showed lower likelihood of severe pneumonia.*

*^b^Pulmonary emphysema positive was denoted as 1, and negative as 0.*

*The Odds Ratio was 8.28 means that patients with pulmonary emphysema showed higher likelihood of severe pneumonia.*

*P value < 0.05, which showed significance.

### Establishment and Verification of Nomogram Model

**FIGURE 3:**
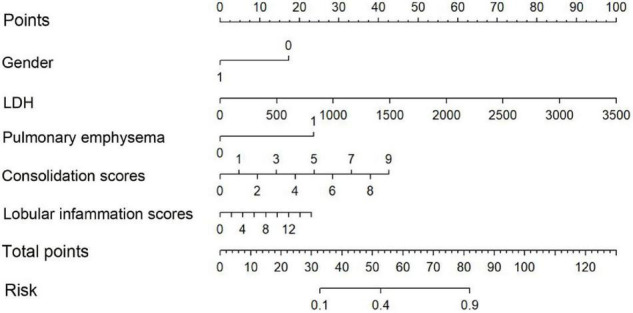
Nomogram. To draw an upward vertical line to the “Points” bar to calculate points. Based on the sum, draw a downward vertical line from the “Total Points” line to calculate the probability of classification of adenoviral pneumonia patient. Male was denoted as 1, and female as 0.

The nomogram (Figure 3) shows that the risk score of gender (1 male, 0 female) was 18 for females, and the risk score for pleural effusion was 24. The risk score of LDH value, consolidation score, and lobular inflammation score varied with children’s glands. The actual value of patients with viral pneumonia increased.

**FIGURE 4:**
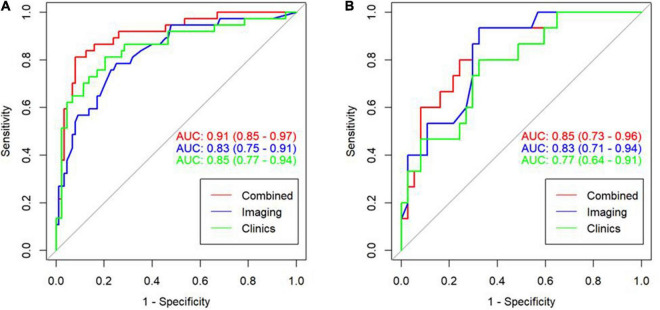
ROC curve for the three model in the training cohort with an AUC of 0.91, 0.83, and 0.85, respectively **(A)**. ROC curve for the three model in the validation cohort with an AUC of 0.85, 0.83, and 0.77, respectively **(B)**.

We have established three different models: clinical model, imaging model, and clinical + imaging combined model (Table 3). The results show that the AUC of the clinical model, imaging model, and combined model in the training set was 0.85, 0.83, and 0.91, respectively (Figure 4A); The AUCs of the clinical model, imaging model, and combined model of the validation set were 0.77, 0.83, and 0.85, respectively (Figure 4B).

**TABLE 3:** Accuracy and predictive value between three models.

	AUC	95%CI	Accuracy	Sensitivity	Specificity	PPV	NPV
**Training Cohort**							
Clinic model	0.85	0.77–0.94	0.80	0.81	0.79	0.63	0.91
Imaging model	0.83	0.75–0.91	0.76	0.78	0.75	0.59	0.89
Combined model	0.91	0.85–0.97	0.86	0.84	0.87	0.74	0.93
**Validation Cohort**							
Clinic model	0.77	0.64–0.91	0.69	0.47	0.78	0.47	0.78
Imaging model	0.83	0.71–0.94	0.73	0.53	0.81	0.53	0.81
Combined model	0.85	0.73–0.96	0.77	0.57	0.90	0.80	0.76

*AUC area under the curve; CI confidence interval; NPV negative-predictive value; PPV positive-predictive value.*

**FIGURE 5:**
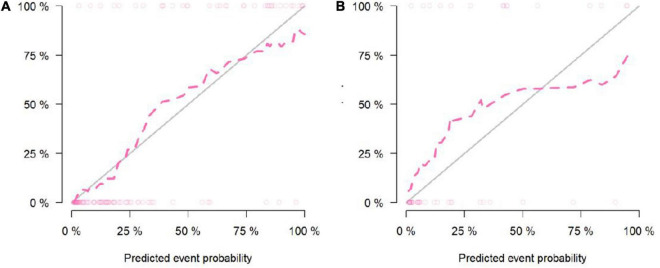
Calibration curve of the Nomogram model in the training cohort **(A)**. Calibration curve of the Nomogram model in the validation cohort **(B)**.

According to the calibration graphs of the training queue (Figure 5A) and the verification queue (Figure 5B), the standard curve in the calibration graph fits well with the predicted calibration curve, and the degree of calibration is good. Hosmer test *P*-value is greater than 0.05, and there is no statistical difference.

**FIGURE 6:**
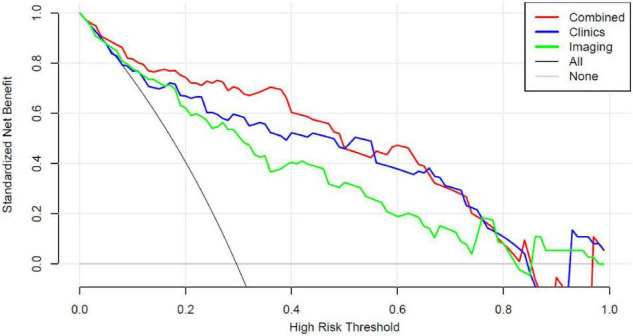
Decision curve analysis (DCA) for the Nomogram model in validation cohort. Compared to other models, the combined Nomogram model, showing the highest area under the curve, is the optimal decision making for maximal net benefit in prediction of severe adenoviral pneumonia.

The analysis of clinical decision curve (Figure 6) shows that the nomogram prediction model has good predictive ability, making appropriate clinical decisions for early severe childhood adenovirus patients. The combined model can bring the greatest clinical benefit to patients.

## Discussion

Human adenovirus (HAdV) was first discovered in 1953 by Rowe and colleagues. It is a non-enveloped double-stranded DNA virus belonging to the genus human mammary adenovirus. They are common pathogens in children and are responsible for various diseases (10). Among the 90 AdV serotypes, 55 are known to cause human diseases, of which AdV3, 4, 7, and 14 are the most common types that cause respiratory disease outbreaks (11). However, in most cases, HAdV is associated with mild to moderate disease, some patients may still develop life-threatening diseases, especially those with a weakened immune system (12). Adenovirus is primarily transmitted through air droplets. Infants, children, the elderly, and people with weakened immune systems are more likely to be infected and develop severe pneumonia (13). Infants and young children are the main populations, and the incidence usually occurs under the age of 2. The severe group in this study had a younger onset age, with an average age of 12 months, which was significantly lower than the mild group (*P* < 0.001). Children in the severe group weighed significantly less than those in the mild group (*P* < 0.001). There are more boys than girls in this study population, which is consistent with the results of other scholars (14, 15). In this study, the proportion of girls who progressed to severe illness was significantly higher than boys, which may be related to the girls’ low weight, nutritional status, and weak immunity. We also cannot rule out the possibility of bias due to the small sample size.

All patients enrolled in this study suffered from fever. The average body temperature of the mild group was 37.1°C (36.6, 38.3), which was lower than the severe group’s 37.9°C (37.0, 38.7); coughing is the most common symptom in patients with adenovirus pneumonia in this group of cases, but it is a non-specific symptom. Fever is also a prominent symptom of this group of cases, and it is mostly a high fever. Adenovirus pneumonia is one of the few viral types of pneumonia that can cause an increase in white blood cell count. There was no significant difference in heat duration between the mild and severe groups, which may be related to the fact that we only counted the data within 3 days of the admission.

The laboratory test indexes L/N ratio, LDH, ALT, AST, CK-MB, and ADV DNA viral load of the mild group were significantly lower than those of the severe group, which may be related to the weak immunity of younger children; another possible mechanism is that adenovirus may have triggered a cytokine storm (9). The levels of AST and LDH in the severe group were significantly higher than those in the mild group, consistent with the findings of Huang et al. (15, 16). Children with severe adenovirus pneumonia may have pulmonary ventilation disorders, resulting in hypoxemia, severe microcirculation disorder, insufficient tissue perfusion, and abnormal cell metabolism. At the same time, pathogenic microorganisms and various inflammatory factors cause various levels of damage to hepatocytes and cardiomyocytes, increasing cell membrane permeability and the concentration of AST and LDH in serum (17), suggesting that adenovirus-induced inflammatory storms are crucial for disease transformation. It is also of great significance.

In this study‘s univariate regression analysis, bronchiectasis, emphysema, bronchial wall thickening, pleural effusion, consolidation score, and lobular inflammation score in the chest low-dose CT findings high-risk factors for severe adenovirus pneumonia, while multivariate regression analysis found that, Emphysema, consolidation score, and lobular inflammation score were independent risk factors. Shen et al. (18) found that children with adenovirus pneumonia with pleural effusion had more severe clinical symptoms, consistent with our research results. Adenovirus and inflammatory mediators cause bronchi and bronchiole mucosal edema, hyperemia, neutrophils, lymphocytes, and other inflammatory cell infiltration around the bronchi and bronchiole and the wall of the tube, resulting in thickening of the bronchial tube wall and necrosis of the bronchial and bronchial mucosa. Necrosis obstructs the lumen as it falls off, and at the same time, mucus secretion increases and obstructs the lumen, resulting in emphysema. The centripetal distribution of lung consolidation after adenovirus pneumonia is closely related to its pathological changes. The lung parenchyma is affected by adenovirus infection, spreading through the small and medium bronchus along the airway. When the lesion involves the bronchioles, it quickly spreads to the alveoli, causing the formation of inflammatory substances. Pulmonary consolidation suggests that many lesions and severely impaired lung function are the fundamental factors leading to severe adenovirus pneumonia.

Although immunofluorescence tests or nucleic acid tests are the standards for clinical diagnosis of adenovirus infection, its application still has some limitations. Not all medical institutions can carry it out. Chest CT imaging is an effective auxiliary diagnosis method that has been widely conducted. Experienced radiologists generate semantic features of different types of information about pneumonia from CT images of patients, such as lobular inflammation, consolidation, mosaic sign, bronchial wall thickening, bronchiectasis, emphysema, pleural effusion, etc. These features can provide useful information for the prognosis and diagnosis of pneumonia. These unique features can be qualitative descriptors, called semantic features that describe the shape and internal structure of the lesion and are scored by radiologists (19–21). They are classified depending on the radiologist’s visual assessment, limiting the lesion’s description to the range visible with the naked eye (22–24).

Semantic features have been used for the first time to construct monographs in this study, especially the consolidation scores and lobular inflammation scores have been used to construct a predictive model for severe childhood adenovirus pneumonia. Compared with imaging omics features, quantitative semantic features analysis is simpler and easier. First, if a large number of inflammatory infiltrations occur on both sides of the lung, it is challenging to outline the area of interest on the CT image accurately, and some patients with mild adenovirus pneumonia have no apparent abnormalities on the CT scans. Secondly, semantic features do not require deep learning software and analysis packages for complex data analysis and processing and construct monographs. The results are stable and reliable, and the clinical diagnosis value is great. The combined model has the highest diagnostic efficiency and can assist clinical diagnosis and prediction. It can be promoted in the majority of primary hospitals.

To construct this prediction model, we list strict including and excluding criteria. All the patients including were required to be diagnosed as mild adenoviral pneumonia at the first 3 days of admission. Therefore, 92 patients were excluded for the diagnosis of severe adenoviral pneumonia at that time. Meanwhile, as one of the best pediatric centers in our province, most patients with mild adenoviral pneumonia in our hospital have a favored prognosis, and only a rather small part of them might convert to severe adenoviral pneumonia. This prediction model shows that the image model has an AUC of 0.83 in the training set and 0.83 in the test set; the AUC of the clinical model in the training set is 0.85, and the AUC in the test set is 0.77; the image + clinical combined model has an AUC of 0.91 in the training set. The centralized AUC is 0.85. The image feature has a large weight and contribution value in this prediction model, with objective indicators and strong stability. The calibration curve in the calibration graph closely fits the prediction calibration curve good. The analysis of the clinical decision curve shows that the nomogram prediction model has an excellent predictive ability, which helps to make appropriate clinical decisions for early severe childhood adenovirus patients and has crucial clinical guidance significance.

This study has several limitations. First, the samples size of this study is limited. It is a single-center retrospective study. Only patients with adenovirus pneumonia undergoing CT examination were selected. The results may have certain deviations. For future studies, it will be necessary to increase the sample size and conduct long-term multi-center clinical research.

## Conclusion

The prediction model based on chest low-dose CT image characteristics and clinical characteristics has relatively clear predictive value in distinguishing mild adenovirus pneumonia from severe adenovirus pneumonia in children. All the selected features of this prediction model are objective and easy-obtained variables, and our results might provide a new method for early clinical prediction of the outcome of adenovirus pneumonia in children.

## Data Availability Statement

The raw data supporting the conclusions of this article will be made available by the authors, without undue reservation.

## Ethics Statement

Written informed consent was obtained from the individual(s), and minor(s)’ legal guardian/next of kin, for the publication of any potentially identifiable images or data included in this article.

## Author Contributions

PL designed the research study. LX and SZ performed the research. HL contributed to the new reagents and analytic tools. PL and YH analyzed the data and wrote the manuscript. All authors have read and approved the final manuscript.

## Conflict of Interest

HL was employed by the company GE Healthcare. The remaining authors declare that the research was conducted in the absence of any commercial or financial relationships that could be construed as a potential conflict of interest.

## Publisher’s Note

All claims expressed in this article are solely those of the authors and do not necessarily represent those of their affiliated organizations, or those of the publisher, the editors and the reviewers. Any product that may be evaluated in this article, or claim that may be made by its manufacturer, is not guaranteed or endorsed by the publisher.
